# A Data-Driven OBE Magnetic Interference Compensation Method

**DOI:** 10.3390/s22207732

**Published:** 2022-10-12

**Authors:** Yizhen Wang, Qi Han, Dechen Zhan, Qiong Li

**Affiliations:** Faculty of Computing, Harbin Institute of Technology, Harbin 150001, China

**Keywords:** aeromagnetic survey, OBE interference compensation, LSTM network

## Abstract

Aeromagnetic compensation is a technology used to reduce aircraft magnetic interference, which plays an important role in aeromagnetic surveys. In addition to maneuvering interferences, the onboard electronic (OBE) interference has been proven to be a significant part of aircraft interference, which must be reduced before further interpretation of aeromagnetic data. In the past, most researchers have focused on establishing linear models to compensate for OBE magnetic interference. However, such methods can only work using accurate reference sensors. In this paper, we propose a data-driven OBE interference compensation method, which can reduce OBE interference without relying on any other reference sensor. This network-based method can integrally detect and repair the OBE magnetic interference. The proposed method builds a prediction model by combining wavelet decomposition with a long short-term memory (LSTM) network to detect and predict OBE interference, and then estimates the local variation of the magnetic field to remove the drift of the interference. In our tests, we construct 10 semi-real datasets to quantitatively evaluate the performance of the proposed method. The F1 score of the proposed method for OBE interference detection is over 0.79, and the RMSE of the compensated signal is less than 0.009 nT. Moreover, we also test our method on real signals, and the results show that our method can detect all interference and significantly reduce the standard deviation of the magnetic field.

## 1. Introduction

Aeromagnetic surveys are a way of measuring the Earth’s magnetic field using a magnetometer mounted on an aircraft. Because of their low cost and high efficiency, aeromagnetic surveys are widely used in archaeological surveys, geological research, mineral exploration, and so on [1]. Aeromagnetic compensation is a technique for eliminating the interference of aircraft and is a key part of aeromagnetic surveys. The most widely used compensation model, called the T-L model, was proposed by Tolles and Lawson. They divided the aircraft interference field into three parts: constant magnetic field, induced magnetic field, and eddy current field, and established a linear regression model to estimate these interferences [2]. However, in practical applications, some onboard electronic (OBE) systems will generate magnetic interferences (called OBE interferences in this paper), which is not described in the T-L model. With the improvement of the accuracy of magnetic field measurement and aeromagnetic compensation, the influence of OBE interference becomes more and more obvious. Therefore, it is very important to continuously monitor and compensate for the OBE interference in aeromagnetic exploration.

At present, there are not many studies on OBE interference compensation. These methods always rely on reference sensors, such as current/voltage sensors and reference magnetometers. In [3,4,5], the current are voltage are measured by corresponding sensors and used to estimate the OBE magnetic interference according to the assumption that the OBE magnetic interference is proportional to the current or voltage. However, there are some problems with this kind of method: (1) The ON/OFF of OBE devices may not be an instantaneous operation [6], during the switching process, the interference is very hard to be well compensated without a very good synchronization between the current/voltage sensor and the magnetometer. (2) On the ground, the ambient magnetic field is too complex and noisy, and the calibration parameters calculated on the ground are hard to be made accurate. The method proposed in [7] is different from the above methods. The authors use a reference magnetometer to adaptively estimate the OBE magnetic interference. This method is particularly effective for time-varying interference with unknown signal characteristics. However, there is also a problem: this method can only handle the case that there is only one interference source, but there are more interference sources on aircraft. Moreover, there is a common problem with these two kinds of methods relying on reference sensors: the installation of reference sensors is difficult, and they must meet Civil Aviation requirements.

In [8], the authors propose a method without reference sensors (called the COOE method in this paper). In this work, the OBE magnetic interference is detected according to the difference between the variances in the magnetic field in the adjacent windows. Then the interference is roughly compensated using linear interpolation. The flaw of this method is that some parameters, such as the sliding window length and the detection threshold, should be manually adjusted carefully to avoid lots of missing alarms. Moreover, the linear interpolation compensation misses magnetic details.

In our opinion, the method of time series processing can be used to detect and compensate for the OBE interference. In recent years, methods based on neural networks, especially long short-term memory (LSTM), have been widely used in context anomaly detection. Since aeromagnetic data are stored in chronological order [9], it can be regarded as a time series. Meanwhile, the OBE interferences can be regarded as context-dependent anomalies. Hundman et al. [10] used LSTM networks to detect anomalies in spacecraft data and proposed a dynamic threshold segmentation method based on past data. Markus Thill et al. [11] proposed an unsupervised ECG anomaly detection method based on stacked LSTM. The anomalies in ECG sequences can be detected by predicting normal sequence behavior and establishing a statistical model of normal behavior prediction error. In [12], the LSTM and Bi-LSTM models are generated to identify rice crops using a Sentinel-1 time series. Ding et al. [13] used an LSTM model to detect errors in industrial manipulator systems. In the remote sensing field, Sun et al. applied LSTM to crop yield prediction [14], and Wang et al. proposed a land cover classification and supervision framework based on LSTM [15].

To avoid the limitations of OBE interference compensation methods with reference sensors, we proposed a data-driven method without any reference sensors. This method is more accurate and reliable than other data-driven methods. We use the LSTM network to identify OBE interference and design a pipeline to repair it. Compared with the COOE method, our proposed compensation method can reduce OBE interference while preserving the details of magnetic field data. To quantitatively evaluate the proposed method, 10 semi-real datasets are constructed using real measured magnetic fields and simulated interferences, each of which contains about 10% OBE interferences. We also test the proposed method in real data and compare it with the COOE method.

In this work, we propose an integrated method to detect and repair the OBE magnetic interference without relying on any reference sensor. To detect the OBE magnetic interference, we propose an LSTM-based network to predict the normal magnetic field and calculate an adaptive threshold of the error between the prediction result and the measured magnetic field. Before that, we use the maximum overlap discrete wavelet transform (MODWT) to decompose the magnetic field into multi-resolution terms, which makes the prediction more accurate. After the detection, we analyze two typical OBE interference types and propose an algorithm to repair them using the mentioned prediction result and the local signal variation. In addition, we also utilize a Gaussian kernel convolution to remove the trend term, which can be embedded into a network and improve the model generalization.

The organization of this paper is as follows. The proposed method is described in Section 2. Section 3 discusses the experimental details, including dataset preparation, model parameters, training configuration, and evaluation metrics. The results and discussion are provided in Section 4. The conclusions are summarized in Section 5.

## 2. Methodology

### 2.1. Background

An aeromagnetic survey usually refers to the collection of geomagnetic signals using aircraft installed with a high-precision magnetometer. The aircraft is always equipped with various OBE devices, such as air conditioning, beacon light, radio, and so on [6,16]. When these devices are working, they produce DC currents, which can generate a magnetic field [6]. These magnetic fields are projected onto the geomagnetic field and are captured by the scalar magnetometer. In this case, the OBE interference can be described by Biot–Savart law and can be viewed as proportional to the current [3,4]. When the device is operating for a long time, OBE interference usually presents as a long-term magnetic field shift. When the device is switched frequently, such as the beacon light continuously sends a pulsed current [17], OBE interference also appears as a pulse-like signal. All these magnetic interferences are named OBE interferences. Without corresponding compensation, such interferences will disturb magnetic field analysis. Therefore, it is very important to compensate for the OBE interference in aeromagnetic surveys.

There are two typical forms of OBE interferences. One type is short-time, peaky, and usually periodic. This kind of interference is called short-term interference, such as interference caused by the beacon light or strobe light. The other type of interference changes quickly at the beginning and end, like short-term interference, but lasts longer. This kind of interference is called long-term interference, such as interference from the radio or rudder motor.

**Figure 1:**
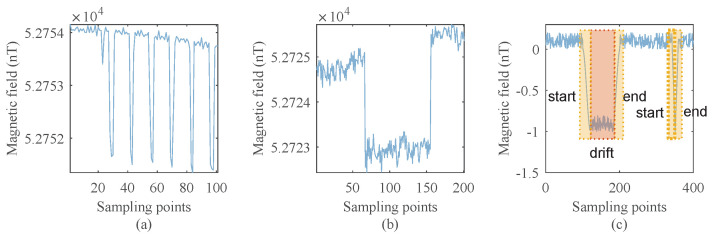
Typical OBE generated by the software interferences: (**a**) the short-term interference, (**b**) the long-term interference, and (**c**) the description of interferences.

In Figure 1, two typical forms of magnetic interference are given. Figure 1a is the short-term interference with peaks. Figure 1b is the long-term interference with data drift.

For the two types of OBE interferences, we give a brief description in Figure 1c, and use light yellow boxes to mark the start and end positions of OBE interferences. In our work, the interferences at the start and end positions are named anomalies, and the part between the start and end positions is named the drift. As can be seen from the figure, these two types of interferences are in the same form at the start and end positions; the difference is there is a data drift in the long-term type. Therefore, we can adopt a unified approach to deal with the two kinds of interference and then deal with the data drift separately.

**Figure 2:**
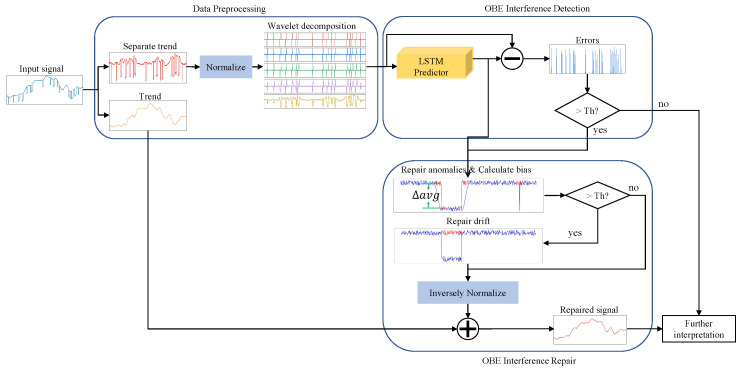
Overview of method structure.

Based on the above analysis, we propose an unsupervised automatic OBE interference compensation framework, as shown in Figure 2. First, the magnetic field data is detrended, normalized, and decomposed using a wavelet. These procedures are collectively called preprocessing. Then we use the LSTM network to model and predict the magnetic field data and detect interferences by comparing the errors between the prediction and the input with an adaptive threshold based on extreme value theory (EVT). With the detection results, we repair the interference anomalies with the predictions and then repair the data drift with calculated bias. Finally, the repaired results are superimposed with the magnetic field trend to obtain the final compensated results.

### 2.2. Data Preprocessing

#### 2.2.1. Trend Separation

The Earth’s magnetic field has a large magnitude of about 50,000 nT [9] and wide range of variation. The variation comes from the sum of diurnal variations, micropulsations, magnetic storms, and long-term variations [18]. Due to the above influence, the variation in the Earth’s magnetic field is complex, and its variation range can reach several hundred nanoteslas in a period of time [9,19]. However, the magnitude of OBE interference is very small. For example, a beacon light can only cause magnetic interference of about 0.25 nT, which is far less than the variation of the Earth’s magnetic field. Therefore, the large variations of the Earth’s magnetic field should be reduced.

As a low-pass filter, a Gaussian filter can extract the low-frequency part of the signal [20]. We take the extracted low-frequency part as the extraction trend and reduce the influence of variations of the Earth’s magnetic field and enhance the interference characteristics by subtracting this part. In addition, since the filter is actually implemented by convolution, this part can be directly embedded into the network.

The Gaussian kernel is expressed as [21]

(1) $$g\left(s,\sigma_{g}\right)=\frac{1}{\sqrt{2\pi }\sigma_{g}}e^{-\frac{s^{2}}{2\sigma_{g}^{2}}},$$

where $\sigma_{g}$ is the standard deviation of the Gaussian function, and *s* is the magnetic field value of a sampling point.

For the measured magnetic field $S_{Total}$, the trend obtained after filtering is $S_{Trend}=conv\left(S_{Total},g\right)$, where conv represents the one-dimensional convolution operation. The smoothness of the trend can be adjusted by changing the $\sigma_{g}$ value. Empirically, the $\sigma_{g}$ is set as 50. The magnetic field after removing the trend is expressed as $S=S_{Total}-S_{Trend}$.

#### 2.2.2. Normalization

The data should be normalized into the network to obtain better performance. Because it is hard to give a suitable minimum or maximum value, we use z-score normalization as follows:

(2) $$X=\frac{S-\mu_{s}}{\lambda \sigma_{s}},$$

where the standardized value is X, the input data are S, the average of the input data is $\mu_{s}$, and the standard deviation of the input data is $\sigma_{s}$.

To keep the value range as close to [−1,1] as possible, we bring λ in to scale $\sigma_{s}$. In our work, λ is experimentally set as 4.

#### 2.2.3. Wavelet Decomposition

Wavelet transform often plays a positive role in time series prediction models [22,23,24]. As stated in [25], wavelet transform is an adaptive time-frequency domain analysis method that can effectively deal with non-stationary time series and non-Gaussian noise and is an effective data enhancement method. For complex sequences, combining wavelet transform with a prediction model has been a widely used data enhancement method, which can effectively improve the accuracy of prediction results [26]. In addition, wavelet transform has a better processing effect for non-stationary signals such as magnetic field measured by a magnetometer [27]. Therefore, based on the good time-frequency localization ability of the wavelet [22], we use wavelet decomposition to decompose the magnetic field data into different scales and obtain the near-periodic expression. We feed signals of different scales into the LSTM network to obtain more accurate prediction results.

In this paper, the MODWT is adopted to process a magnetic field with the trend removed. The MODWT is a time-shift invariant, redundant, and non-orthogonal transformation method. Compared with other wavelet transform methods, the MODWT has the following advantages [25]: (1) the ability to handle any sample size; (2) increased coarse scale resolution; (3) a more asymptotically efficient estimation of wavelet square difference than DWT; (4) it can deal with non-stationary time series and non-Gaussian noise more effectively. More information about MODWT can be found in [22,26,28].

Here, MODWT [29,30] is used to analyze the time series. When the MODWT is applied to the time series X, the wavelet and the scaling coefficients of level *j* are

(3) $${\tilde{W}}_{j,t}=\sum_{l=0}^{L_{j}-1}{\tilde{h}}_{j,l}X_{t-lmodN},$$

(4) $${\tilde{V}}_{j,t}=\sum_{l=0}^{L_{j}-1}{\tilde{g}}_{j,l}X_{t-lmodN},$$

where ${\left\{{\tilde{h}}_{j,l}\right\}}_{l=0}^{L_{j}-1}$ and ${\left\{{\tilde{g}}_{j,l}\right\}}_{l=0}^{L_{j}-1}$ are the wavelet and scaling filters of level *j*, respectively. The filter width is expressed as

(5) $$L_{j}=\left(2^{j}-1\right)\left(L_{1}-1\right)+1,$$

where $L_{1}$ is the width of the unit-level Daubechies wavelet coefficient. In order to avoid the influence caused by the boundary conditions of the wavelet transform, we first remove the $L_{j}$ wavelet and the scale coefficients (determined by Equation (5)), and obtain the “boundary correction” wavelet and scaling coefficients [26].

### 2.3. OBE Interference Detection

In this section, we design an unsupervised OBE interference detection algorithm. The algorithm is trained on normal magnetic field data without OBE interference and outputs the prediction error. The Extreme Value Theory (EVT) method is used to set the threshold automatically to detect OBE interference.

#### 2.3.1. Magnetic Field Predictor Based on LSTM

The LSTM network was first proposed by Hochreiter and Schmidhuber as a variant of RNN [15]. The LSTM can make full use of the historical information and time dependence of modeling signals [31]. It allows subsequent states at different time intervals to be stored by regularly connecting hidden layer nodes, where parameters are shared between different parts of the model. Many studies have proven that LSTM is very suitable for processing time series data [10,32,33,34]. The basic structure of the LSTM includes an input gate, forgetting gate, output gate, and internal storage unit. In this paper, we adopted the LSTM formula proposed by Graves et al. [35], and the key formulas are expressed as

(6) $$\begin{array}{cc} \; & f_{t}=\sigma \left(W_{f}\left[h_{t-1},x_{t}\right]+b_{f}\right),\\ \; & i_{t}=\sigma \left(W_{i}\left[h_{t-1},x_{t}\right]+b_{i}\right),\\ \; & {\tilde{C}}_{t}=tanh\left(W_{C}\left[h_{t-1},x_{t}\right]+b_{C}\right),\\ \; & C_{t}=f_{t}*C_{t-1}+i_{t}*{\tilde{C}}_{t},\\ \; & o_{t}=\sigma \left(W_{o}\left[h_{t-1},x_{t}\right]+b_{o}\right),\\ \; & h_{t}=o_{t}*tanh\left(C_{t}\right), \end{array}$$

where the subscript t−1 is the previous time, and *t* is the current time. The operator ∗ represents the Hadamard product. Two activation functions are used, the hyperbolic tangent function tanh(·) and the sigmoid function σ(·). The different weight matrices *W* and deviation *b* are the parameters to be trained. The forgetting gate *f* determines how much information is forgotten in the output $h_{t-1}$ and the current time input $x_{t}$. Similarly, the input gate *i* determines which values will be updated. *C* is the updated state for the current cell. The output gate *o* determines which parts of the cell state will be output. Finally, the updated hidden state is *h*.

**Figure 3:**
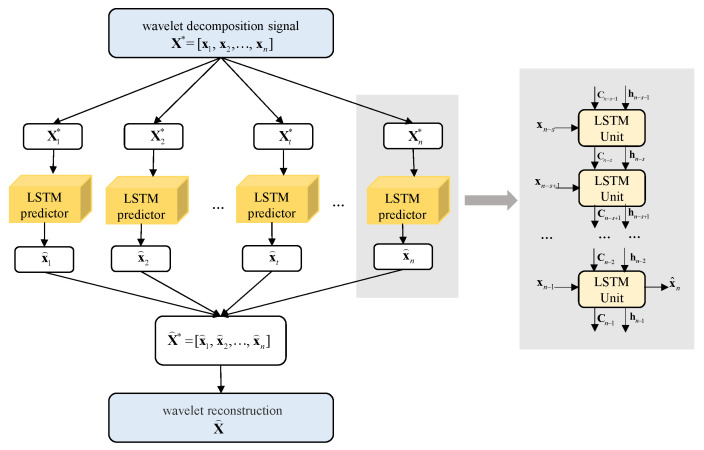
Structure of the LSTM.

Suppose that the sequence of the preprocessed magnetic field is $X^{*}=\left[x_{1},x_{2},\ldots ,x_{n}\right]$, where $x_{i}\in R^{m}\left(i=1,\ldots ,n\right)$ is the *m*-dimensional component of magnetic field data of the *i*th sampling point obtained after wavelet decomposition, and *n* is the number of points. Assuming the embedding time step of LSTM is *s* and the predicting step length l=1. For the magnetic field of the *t* sampling point, the sequence $X_{t}^{*}=\left[x_{t-s},x_{t-s+1},\ldots ,x_{t-1}\right]$ with length *s* is used as the input of the LSTM network to obtain the predicted value ${\hat{x}}_{t}$. At time t−1, the structure of the multidimensional LSTM is shown in Figure 3 below.

The predicted sequence is ${\hat{X}}^{*}$, $\hat{X}$ obtained using wavelet reconstruction to ${\hat{X}}^{*}$.

#### 2.3.2. Adaptive Threshold Based on EVT

Predictors are trained on normal magnetic data without OBE interference. The output prediction error $E_{t}$ is defined as the square of the difference between the observed value and the corresponding predicted value at time *t* and is expressed as

(7) $$E_{t}={\left(X_{t}-{\hat{X}}_{t}\right)}^{2}.$$

All prediction errors in the training dataset are expressed as a vector $E=[E_{1},E_{2},\ldots ,E_{n}]$. We use the Peak Over Threshold (POT) method based on EVT to adaptively adjust the detection threshold. The EVT is a statistical theory aiming to find patterns of extreme values, which are usually at the tail of probability distributions [36]. The advantage of EVT is that there is no need to make assumptions about data distributions when looking for extreme values [37,38]. The POT is the second theorem in EVT, and the basic idea of that is to use the generalized Pareto distribution (GPD) with parameters to fit the tail of the probability distribution. The GPD function is as follows:

(8) $${\bar{F}}_{th}\left(e\right)=P\left(E-th>e|E>th\right)∼{\left(1+\frac{\gamma e}{\sigma }\right)}^{-\frac{1}{\gamma }},$$

where th is the initial threshold, and γ and σ are the shape and scale parameters of GPD. *E* represents the data point in E, and E−th represents the portion that exceeds the threshold th. The values of parameters γ and σ are estimated by maximum likelihood estimation (MLE). Then the final threshold Th is calculated [37].

In the detection process, the prediction error E of the test dataset is used to detect whether there is OBE interference in the test dataset. If a value in E exceeds threshold Th, the corresponding magnetic data are considered anomalies. The detection result can be expressed as $E_{a}=\left\{E_{i}\in E|E_{i}>Th\right\},i=1,2,\ldots ,n$. The detected anomalies D are represented as continuous sequences of $E_{a}\in E_{a}$; the start and end point of *j*th detected anomaly $D_{j}$ are denoted as $ano_{begin}^{j}$ and $ano_{end}^{j}$, respectively.

### 2.4. OBE Interference Repair

**Figure 4:**
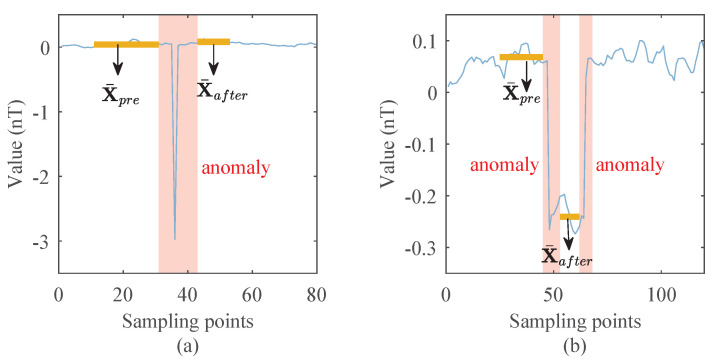
Detection results of two kinds of OBE interferences: (**a**) A detected short-term interference. (**b**) A detected long-term interference. The anomaly segments are marked with a red background.

The mode of OBE interference includes not only spike-like short-term interference but also drift-like long-term interference. The detection results of these two types of OBE interference are shown in Figure 4. The parts where the OBE interference appears and disappears are detected and marked with red backgrounds. These parts (named anomalies) should be corrected. In addition, notice that if the OBE interference is long-term, there is a data drift in the magnetic field, which should also be corrected.

For each detected anomaly, the repair method can be decomposed into two steps: (1) repair anomaly segment with predicted points; (2) if a data drift exists, repair it with the evaluated drift magnitude.

#### 2.4.1. Anomaly Repair

The anomaly segments are detected according to the method in Section 2.3. For each detected anomaly segment, we use the LSTM-based predictor to obtain the predicted value and repair the segment point by point. For each point in this anomaly segment, we obtain the predicted value and update the corresponding point using the LSTM network. The operation is looped until this anomaly segment is processed.

Algorithm 1 shows the detailed steps of anomaly repair.
**Algorithm 1:** Anomaly repair algorithm
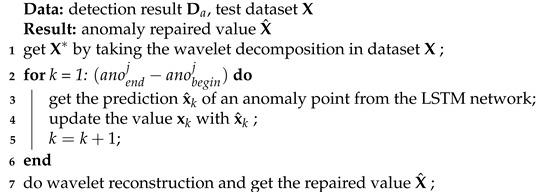


#### 2.4.2. Drift Repair

As can be seen from Figure 4, when there is a drift between the data in front of the anomaly and the data behind the anomaly, the mean value of the data will change significantly. Therefore, the existence of drift can be judged according to the change in the mean value.

Drift repair can be expressed in the following steps:

1. We take the data before and after the anomaly as the basis for determining drift. In order to further reduce the influence of the small trend, we intercept them and get $X_{pre\_cut}$ and $X_{after\_cut}$;
2. We calculate the change in the mean before and after the anomaly and write it as Δavg;
3. We use the Neyman–Pearson (N-P) criterion for $X_{pre}$ to calculate the threshold value $th_{avg}$, and determine whether the drift exists according to the threshold;
4. When drift exists, the repaired data are obtained by summing the predicted value and the difference corresponding to the drift part.

Algorithm 2 shows the detailed steps of drift repair.
**Algorithm 2:** Drift repair algorithm
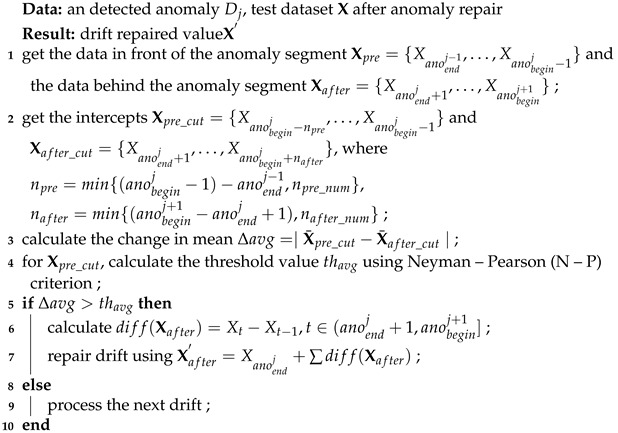


## 3. Experimental Details

### 3.1. Data Preparation

#### 3.1.1. Background Data

The background data were obtained outdoors using an optically pumped cesium vapor magnetometer and a self-made collector. The setup of the collection experiment is the same as our previous work [39]. We set the sampling rate to 10Hz, as is often used in aeromagnetic surveys [40]. The range of the measured magnetic field is about 50,104 to 50,130 nT. We collected magnetic field data for two days, denoted as Day1_Data and Day2_Data, and used them for training and validation, respectively.

#### 3.1.2. Semi-Real Data

**Figure 5:**
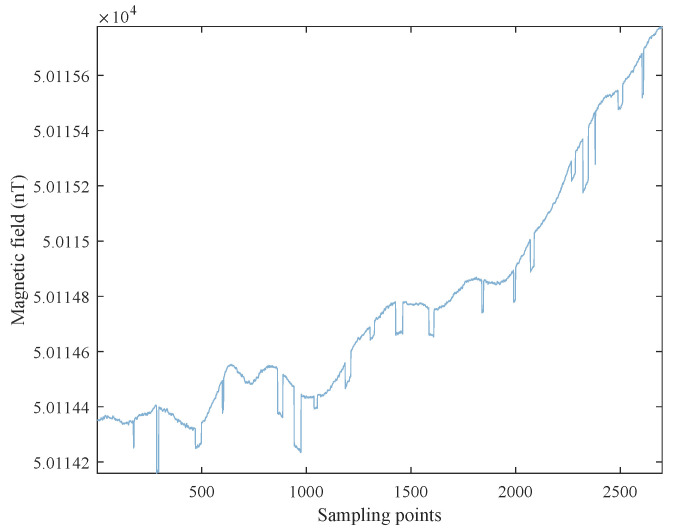
A segment of the semi-real datasets.

In aeromagnetic surveys, it is difficult to obtain a pure magnetic field that can be used as ground truth when OBE interference exists. Therefore, we use semi-real data to quantitatively evaluate the performance of the proposed method. We generate 10 semi-real datasets using the background field and simulated OBE interferences. We divide the background magnetic field Day2_Data into 10 segments of similar length and denote them as C-1 to C-10. For each segment, we randomly select 10% to add simulation OBE interferences. The OBE interference is generated according to the model in [4]. The interference length is 1–40 sampling points, and the intensity is between 0.04nT and 0.2nT. The 10 semi-real datasets are called S-1 to S-10. Figure 5 shows a segment of the semi-real datasets.

#### 3.1.3. Magnetic Field with Real OBE Interferences

To validate the method, we also collected real magnetic field data with OBE interference, as shown in Figure 1. Figure 1a,b are interferences caused by a beacon light and a radio, respectively. The collection equipment consists of a scalar magnetometer and a three-axis vector magnetometer, and the sampling rate is consistent with Section 3.1.1. The interference magnetic field of the beacon light is collected by the scalar magnetometer mounted at the end of the elongated tail. The interference magnetic field of the radio is composed of the vector magnetometer data.

### 3.2. Model Training

#### 3.2.1. Model Parameters

The structure and parameters of the LSTM model are shown in Table 1.

The model consists of two hidden layers, each with 128 cells. We find that this structure provides sufficient capacity to predict the magnetic field well. Increasing the model size provides few benefits. Moreover, the sequence length *s* is set as 32, it provides a good balance between performance and time cost. The model was trained for 200 iterations.

In order to train the LSTM model correctly, the background dataset Day1_Data is divided into an 80% training set and a 20% verification set. The Adam optimizer uses the mean absolute error (MAE) as a loss function. The batch size and learning rate are set as 64 and 0.0001, respectively. For the training step, we use a computer with an Intel Core i7-8700K CPU and an NVIDIA GeForce 1080Ti GPU.

#### 3.2.2. Evaluation Indicators

Since the real datasets lack the ground truth value, we use different evaluation indicators on semi-real datasets and real datasets to evaluate the performance of the proposed method.

For the semi-real datasets, the real label of OBE interference and the pure magnetic field is known, so the detection label and the compensated magnetic field are quantitatively evaluated with the ground truth.

We use the modified range-based precision, recall, F1 score [41,42], and the ROC (Receiver Operating Characteristic) curve to evaluate the interference detection performance. For a dataset, the set of real anomaly ranges is denoted as *R*, $R_{i}$ represents the *i*th real anomaly range, and $N_{r}$ is the number of real anomalies. The set of detected anomaly ranges is denoted as *P*, $P_{i}$ is the *i*th detected anomaly range, and $N_{p}$ is the number of detected anomalies. The $Reward_{existence}$ represents the number of intersections between the detected anomaly ranges and the real anomaly ranges, and the $Reward_{overlap}$ is a function that describes the overlap situation between the detected anomaly ranges and the real anomaly ranges (see [41] for more details). The recall is defined as Equation (9), which colligates the $Reward_{existence}$ and $Reward_{overlap}$ using factor α. In this paper, we set α as 0.5 to treat $Reward_{existence}$ and $Reward_{overlap}$ fairly. The precision is defined as Equation (10), which mainly evaluates the overlap between real anomaly ranges and detects anomaly ranges. The F1 score is defined as Equation (11), which is the harmonic mean of the above two metrics and reflects the robustness of the detection algorithm. The ROC represents the detection ability of the model when the discriminative threshold changes, and the area under the ROC curve (often referred to as AUC) is used to measure the probability that a model can be classified correctly [43].

(9) $$\mathrm{Recall}=\frac{\sum_{i=1}^{N_{r}}\left(\alpha \times Reward_{existence}\left(R_{i},P\right)+\left(1-\alpha \right)\times Reward_{overlap}\left(R_{i},P\right)\right)}{N_{r}},$$

(10) $$\mathrm{Precision}=\frac{\sum_{i=1}^{N_{p}}Reward_{overlap}\left(R,P_{i}\right)}{N_{p}},$$

(11) $$F1\;\mathrm{score}=\frac{2\times \mathrm{Precision}\times \mathrm{Recall}}{\mathrm{Precision}+\mathrm{Recall}}.$$

For the OBE interference repair results, we use the root mean squared errors (RMSE) to evaluate the performance [44,45], which is defined as Equation (12). The RMSE represents the deviation between the repaired results and the clean reference data of all OBE interferences. Therefore, the calculation of RMSE is dependent on the detection results and only includes the repair results of the interference part rather than the complete magnetic field series.

(12) $$\mathrm{RMSE}=\sqrt{\frac{1}{n}\sum_{i=1}^{n}{\left(X_{i}^{{}^{\prime }}-X_{i}^{pure}\right)}^{2}},$$

where $X_{i}^{{}^{\prime }}$ is the *i*th repaired OBE interference, $X_{i}^{pure}$ is the corresponding pure magnetic field without interference, and *n* is the number of anomalies.

For real datasets, we adopted the standard deviation (STD) and improvement ratio (IR) after bandwidth filtering to evaluate the repair results, which are common performance evaluation indexes in magnetic exploration [46,47].

## 4. Results and Discussion

### 4.1. Selection of the Mother Wavelet and Wavelet Decomposition Level

The selection of the mother wavelet and the level of wavelet decomposition will affect the performance of the predictor and then affect the detection and repair effect. In this paper, several orthogonal mother wavelets of the wavelet family are compared, including haar (db1), Daubechies (db2, db3, db4, db5), Symlets (sym3, sym5), and Coiflets (coif1, coif2) [48]. For each mother wavelet, we explore 1 to 5 levels of decomposition, denoted as L1 to L5, respectively. At the same time, we also test the case without using wavelet decomposition, denoted as L0. The selection of mother wavelet and decomposition levels is based on the prediction results in the pure magnetic field. We use RMSE to measure the prediction error, and the lower RMSE value represents that the predictor performs better. We use Day_1 Data for training and test each combination of mother wavelet and decomposition level on C-1 to C-10 and calculate the average RMSE for 10 datasets, as shown in Table 2.

The test results show that using the haar wavelet with four decomposition levels can achieve the best prediction performance. The haar wavelet has three advantages: (1) it can capture the fluctuations between adjacent data, (2) it does not have an aliasing problem, and (3) it can express the low-frequency features well [49]. The main components of the geomagnetic field are also mostly distributed in low frequency [50], so using the haar wavelet decomposition is conducive to the LSTM network for learning geomagnetic field characteristics.

### 4.2. Semi-Real Dataset Test Results

The detection results on the semi-real dataset are shown in Table 3. We compare the proposed detection method with the COOE method. Note that the parameters of the COOE method are set to the maximum of the F1 score of each dataset.

As shown in Table 3, our method performs better in three indicators. The recalls of our method are larger than 0.92 in all datasets and equal to 1 in 7 datasets, which indicates that our method can detect almost all anomalies. However, the recalls of the fine-tuned COOE algorithm are below 0.7 in all datasets, mostly in the range of 0.5 to 0.64, which means that it misses a lot of anomalies. The precisions of our method are significantly higher than the COOE method, which indicates that our method mislabels fewer normal cases. The precisions of the COOE method are very small because the anomaly length defined in this paper is small, and the detection results of the COOE method often have large biases from real anomalies. Moreover, the higher F1 scores of our method indicate that our method has better comprehensive capacity.

**Figure 6:**
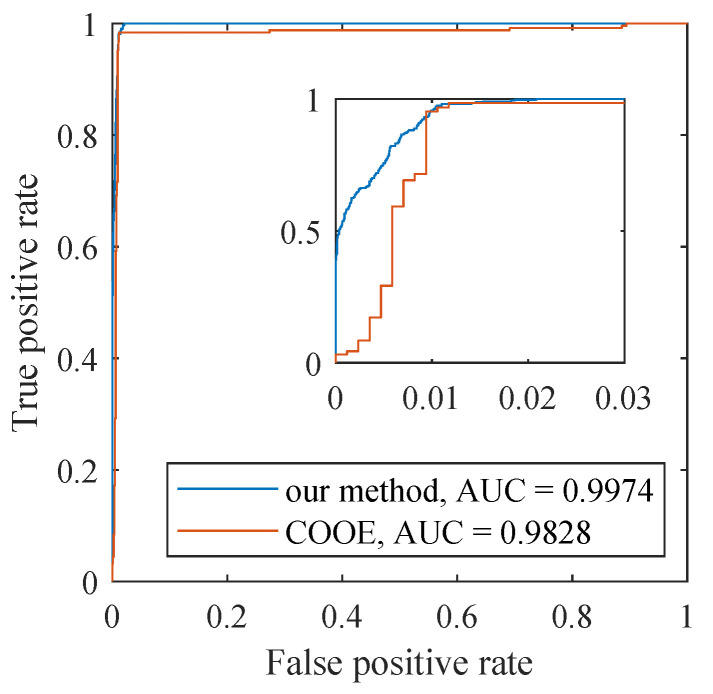
The ROC curves of the two methods in semi-real datasets.

We also compare the ROC curves and calculate the AUCs of the two methods, as shown in Figure 6. The ROCs and AUCs were calculated by counting all detection results from S-1 to S-10. Our method achieves a higher AUC value, which is close to 1, indicating that our method can detect anomalies more accurately.

**Figure 7:**
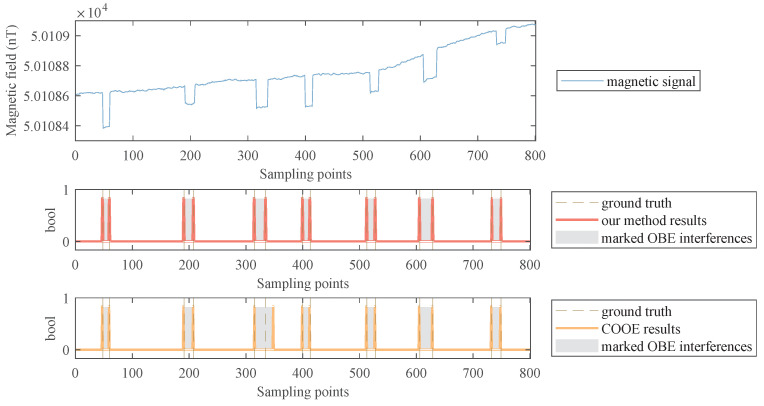
The comparison of detection results in the semi-real data segment.

Figure 7 shows the detection results of a segment of semi-real data. We can find that our method can detect the OBE interference well, almost consistent with the ground truth. The COOE method can also detect all the OBE interferences, but there are some offset points for some OBE interferences. This phenomenon is caused by the fixed-length and non-overlapping sliding window used in COOE. As a note, the fixed window length is hard to pick.

Based on the detection results above, we compare the OBE interference repair performance of the proposed method with that of the interpolation method in COOE on the same datasets. The comparisons of the repair results are shown in Table 4.

As shown in Table 4, the proposed method obtained lower RMSEs in all datasets, indicating that the error between the repaired results of our method and the pure magnetic field without OBE interferences is smaller.

**Figure 8:**
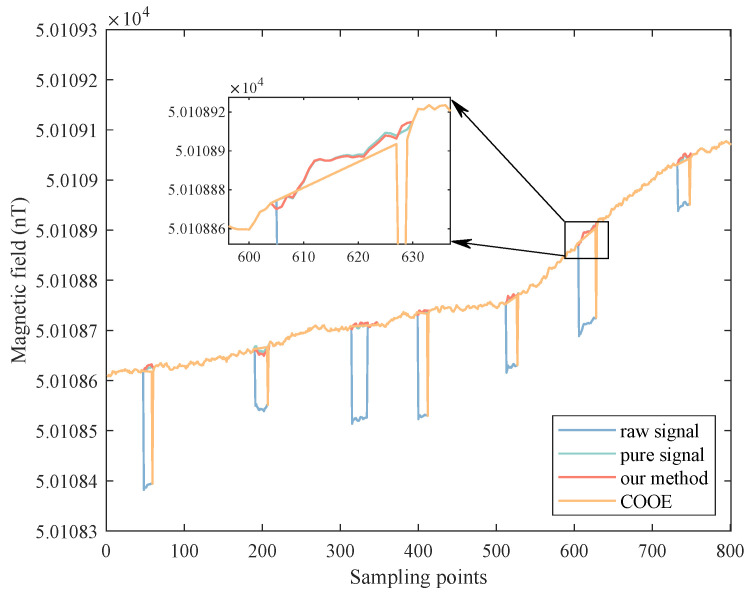
The comparison of repair results in the semi-real data segment.

Figure 8 shows the repair results of the same semi-real data segment. It can be seen that the results of our method are closer to the ground truth. However, due to the deviation of the detection results, the interpolation results obtained by the COOE method are not satisfactory, and many OBE interferences can not be completely removed.

### 4.3. Cross-Validation of Semi-Real Datasets

To verify the generalization of the model, we conduct cross-validation on Day_2 Data. We use the leave-one-out method to carry out 10-fold cross-validation and calculate the detection and repair criteria of 10 validations. For each fold, we select 9 segments from C-1 to C-10 datasets for model training and use the remaining dataset with OBE interferences for verification. For example, for the first fold, we choose C-1 to C-9 as the training set and use S-10 as the test dataset. The final indicators of cross-validation are shown in Table 5. These results are similar to those in Section 4.2 using Day_1 Data as the training set, which proves the good generalization of our model.

### 4.4. Real Datasets Test Results

We verified the effectiveness of the proposed method on the real collected magnetic fields with beacon light and radio interferences; these two datasets are named R-1 and R-2. Since a clean magnetic field cannot be obtained for comparison in real aeromagnetic measurement, we compared the detection results with manual marks and evaluated the magnetic series before and after repair using standard deviation (Std) and improvement ratio (IR).

**Figure 9:**
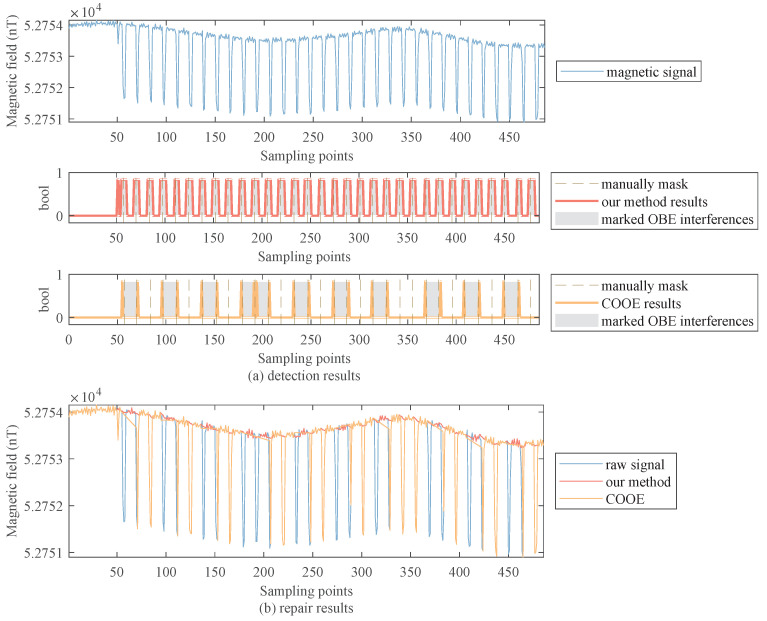
Detection and repair results of the aircraft’s beacon light interferences.

Figure 9a,b are the detection and repair results of beacon light interferences, respectively. From this figure, we find that our method can detect all the interferences and repair them well. In contrast, the COOE method misses some interferences in the detection procedure, which leads to the repaired signal having obvious mutations.

**Figure 10:**
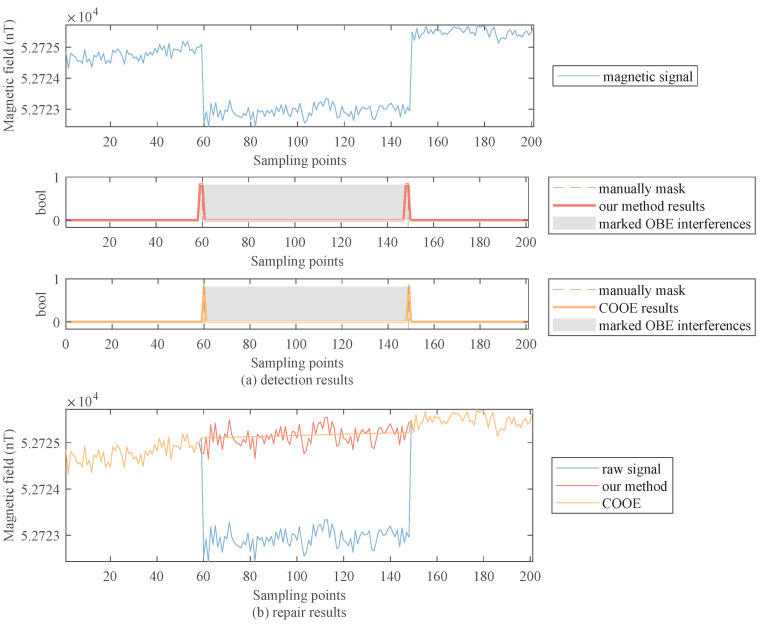
Detection and repair results of the aircraft’s radio interference.

Figure 10a shows the detection results of the aircraft’s radio interference, both methods can detect the interference. Figure 10b shows the repair results of the radio interference. Because the COOE method uses the linear interpolation method to repair, its result completely loses the data characteristics of the magnetic field. Our method can retain the characteristics of the magnetic field data.

Table 6 shows the STDs and IRs of repaired magnetic fields after band-pass filtering. It is a conventional operation in aeromagnetic compensation to perform band-pass filtering before calculating and evaluating STD and IR, and the filter bandwidth is set to 0.1 to 0.6 Hz, according to [51]. Our method achieves smaller STD and larger IR in R-1. The IR of COOE is even less than 1 because the data mutation caused by linear interpolation will produce a large oscillation after filtering. Although the COOE method has a better STD and IR in R-2, the data characteristics of the magnetic field are totally removed. In conclusion, our method can effectively eliminate the current magnetic interference without affecting the characteristics of the magnetic field.

## 5. Conclusions

In aeromagnetic surveys, the interference of OBE magnetic fields is non-negligible. The previous methods often use reference current or magnetic sensors to estimate and remove the OBE interference. In this paper, we propose an unsupervised and unreferenced method to integrally detect and repair them. The detection is determined by an LSTM-based predictor. The threshold of the error between the prediction result and the measured magnetic field is adaptively calculated by the POT algorithm. Moreover, wavelet decomposition is also utilized to improve the prediction accuracy. After detection, based on the prediction result, we design an algorithm to repair the OBE magnetic interference. In contrast with the methods based on interpolation, our method can retain the detailed signal characteristics. In addition, we embed a Gaussian kernel convolution layer into the network, which can detrend the signal and improve the model generalization.

We compare the proposed method with a previous work relying on no reference sensor. On semi-real datasets, it is shown that our proposed method is better than the COOE method in the range-based recall, precision, F1 score, AUC, and RMSE. On real datasets, the results also show that our method can effectively compensate for OBE interferences and increases the improvement ratio. In addition, our method can retain the normal magnetic field characteristics in long-term interference, which ensures the validity of the magnetic field data. 

## Figures and Tables

**Table 1:** Parameters of LSTM model.

Parameters	Configuration
Hidden layers	2
Units in a hidden layer	128
Batch size	64
Input length *s*	32
Optimizer	Adam
Loss function	Mean absolute error

**Table 2:** The RMSEs using different mother wavelets and decomposition levels. **Bold** indicates the best performance.

Mother Wavelet	Decomposition Level					
	L0	L1	L2	L3	L4	L5
haar	0.001780	0.000884	0.000714	0.000696	**0.000688**	0.000716
db2	0.001780	0.003633	0.005849	0.005247	0.004747	0.004378
db3	0.001780	0.003844	0.003943	0.004517	0.003855	0.003084
db4	0.001780	0.005506	0.003690	0.003313	0.001994	0.002576
db5	0.001780	0.005884	0.003094	0.002987	0.003567	0.003157
sym3	0.001780	0.003131	0.003031	0.004244	0.004922	0.004361
sym5	0.001780	0.008126	0.002975	0.003574	0.003958	0.004758
coif1	0.001780	0.002815	0.003041	0.003081	0.006133	0.003810
coif2	0.001780	0.004355	0.003689	0.003688	0.002947	0.002925

**Table 3:** Comparisons of detection results between the proposed method and the COOE method in semi-real datasets.

Datasets	Recall		Precision		F1Score	
	Our Method	COOE Method	Our Method	COOE Method	Our Method	COOE Method
S-1	1.0	0.630303	0.678182	0.042217	0.808234	0.079134
S-2	1.0	0.541667	0.665278	0.061805	0.798999	0.110950
S-3	1.0	0.686275	0.722222	0.173126	0.838710	0.276499
S-4	0.928571	0.607143	0.696000	0.104415	0.795638	0.178185
S-5	1.0	0.603175	0.693651	0.109053	0.819119	0.184724
S-6	1.0	0.506667	0.756410	0.117079	0.861314	0.190206
S-7	1.0	0.535354	0.784314	0.086793	0.879121	0.149370
S-8	1.0	0.699187	0.697619	0.101281	0.821879	0.176932
S-9	0.928571	0.543210	0.732000	0.181519	0.818651	0.272110
S-10	0.952381	0.547619	0.726667	0.044104	0.824352	0.081633

**Table 4:** Comparisons of repair results between the proposed method and the COOE method in semi-real datasets.

Datasets	RMSE	
	Our Method	COOE Method
S-1	0.004714	0.070650
S-2	0.003219	0.071394
S-3	0.004052	0.077023
S-4	0.007610	0.062593
S-5	0.004541	0.060930
S-6	0.005480	0.055922
S-7	0.004271	0.047831
S-8	0.004401	0.080440
S-9	0.008943	0.110222
S-10	0.005094	0.058883

**Table 5:** The average criterions of 10-fold cross-validation.

Fold Number	Criterions				
	Precision	Recall	F1 Score	RMSE	AUC
1	0.750909	1.0	0.857736	0.004047	0.999209
2	0.633333	1.0	0.775510	0.010280	0.999046
3	0.694444	1.0	0.819672	0.003990	0.996489
4	0.668000	0.928571	0.777022	0.007349	0.994278
5	0.677273	1.0	0.807588	0.005208	0.997167
6	0.782051	1.0	0.877698	0.004841	0.996627
7	0.750000	1.0	0.857143	0.004252	0.996927
8	0.788596	0.928571	0.852879	0.010630	0.996776
9	0.698611	0.928571	0.797342	0.007548	0.994613
10	0.690244	0.952381	0.800396	0.006627	0.996710
averages	0.713346	0.973809	0.822299	0.005414	0.996784

**Table 6:** Comparisons of repair results between the proposed method and the COOE method on real datasets.

Datasets	STD		IR	
	Our Method	COOE Method	Our Method	COOE Method
R-1	0.0108	0.2402	20.5823	0.9512
R-2	0.0759	0.0554	5.5604	7.6164

## Data Availability

Not applicable.

## References

[1] Butler D.K. (2005). Near-Surface Geophysics.

[2] Leliak P. (1961). Identification and evaluation of magnetic-field sources of magnetic airborne detector equipped aircraft. IRE Trans. Aerosp. Navig. Electron..

[3] Noriega G., Marszalkowski A. (2017). Adaptive techniques and other recent developments in aeromagnetic compensation. First Break.

[4] Du C., Wang H., Wang H., Xia M., Peng X., Han Q., Zou P., Guo H. (2019). Extended aeromagnetic compensation modelling including non-manoeuvring interferences. IET Sci. Meas. Technol..

[5] Conner C.I., Holmes J.J., Pugsley D.E. (2013). Algorithmic Reduction of Vehicular Magnetic Self-Noise. U.S. Patent.

[6] AARC510 Adaptive Aeromagnetic Real-Time Compensator User’s Guide. www.rmsinst.com/products/aeromagnetic/AARC510.pdf.

[7] Sheinker A., Moldwin M.B. (2016). Adaptive interference cancelation using a pair of magnetometers. IEEE Trans. Aerosp. Electron. Syst..

[8] Abdelhamid B., Elkattan M. (2017). Cancellation of dynamic ON/OFF effects in airborne magnetic survey. Sens. Imaging.

[9] Reeves C. (2005). Aeromagnetic Surveys—Principles, Practice and Interpretation.

[10] Hundman K., Constantinou V., Laporte C., Colwell I., Soderstrom T. Detecting spacecraft anomalies using lstms and nonparametric dynamic thresholding. Proceedings of the 24th ACM SIGKDD International Conference on Knowledge Discovery & Data Mining.

[11] Thill M., Däubener S., Konen W., Bäck T., Barancikova P., Holena M., Horvat T., Pleva M., Rosa R. Anomaly detection in electrocardiogram readings with stacked LSTM networks. Proceedings of the 19th Conference Information Technologies-Applications and Theory (ITAT 2019), CEUR-WS.

[12] Crisóstomo de Castro Filho H., Abílio de Carvalho Júnior O., Ferreira de Carvalho O.L., Pozzobon de Bem P., dos Santos de Moura R., Olino de Albuquerque A., Rosa Silva C., Guimarães Ferreira P.H., Fontes Guimarães R., Trancoso Gomes R.A. (2020). Rice crop detection using LSTM, Bi-LSTM, and machine learning models from Sentinel-1 time series. Remote Sens..

[13] Ding S., Morozov A., Vock S., Weyrich M., Janschek K. (2020). Model-Based Error Detection for Industrial Automation Systems Using LSTM Networks. Proceedings of the International Symposium on Model-Based Safety and Assessment.

[14] Sun J., Di L., Sun Z., Shen Y., Lai Z. (2019). County-level soybean yield prediction using deep CNN-LSTM model. Sensors.

[15] Wang H., Zhao X., Zhang X., Wu D., Du X. (2019). Long time series land cover classification in China from 1982 to 2015 based on Bi-LSTM deep learning. Remote Sens..

[16] Teskey D., Barlow R., Hood P., Lefebvre D., Paterson N., Reford M., Watson D. (1991). Guide to Aeromagnetic Specifications and Contracts.

[17] Burgmann V. (1949). Distance-measuring equipment for aircraft navigation. Proc. IEE-Part III Radio Commun. Eng..

[18] Lee T.N., Canciani A.J. Aerial Simultaneous Localization and Mapping Using Earth’s Magnetic Anomaly Field. Proceedings of the 2019 International Technical Meeting of the Institute of Navigation.

[19] Brennan J.A. (1975). The Effect of Geomagnetic Micropulsations on Mad Systems.

[20] Seddik H., Braiek E.B. (2012). Efficient noise removing based optimized smart dynamic Gaussian filter. Int. J. Comput. Appl..

[21] Hastie T., Tibshirani R., Friedman J.H., Friedman J.H. (2009). The Elements of Statistical Learning: Data Mining, Inference, and Prediction.

[22] Zhu L., Wang Y., Fan Q. (2014). MODWT-ARMA model for time series prediction. Appl. Math. Model..

[23] Wu C., Zhang X., Wang W., Lu C., Zhang Y., Qin W., Tick G.R., Liu B., Shu L. (2021). Groundwater level modeling framework by combining the wavelet transform with a long short-term memory data-driven model. Sci. Total Environ..

[24] Liu B., Zhang L., Wang Q., Chen J. (2021). A novel method for regional NO_2_ concentration prediction using discrete wavelet transform and an LSTM network. Comput. Intell. Neurosci..

[25] Wen Q., Sun L., Yang F., Song X., Gao J., Wang X., Xu H. (2020). Time series data augmentation for deep learning: A survey. arXiv.

[26] Quilty J., Adamowski J. (2018). Addressing the incorrect usage of wavelet-based hydrological and water resources forecasting models for real-world applications with best practices and a new forecasting framework. J. Hydrol..

[27] Kumar P., Foufoula-Georgiou E. (1997). Wavelet analysis for geophysical applications. Rev. Geophys..

[28] Mouatadid S., Adamowski J.F., Tiwari M.K., Quilty J.M. (2019). Coupling the maximum overlap discrete wavelet transform and long short-term memory networks for irrigation flow forecasting. Agric. Water Manag..

[29] Daubechies I. (1992). Ten Lectures on Wavelets.

[30] Percival D.B., Walden A.T. (2000). Wavelet Methods for Time Series Analysis.

[31] Yu Y., Si X., Hu C., Zhang J. (2019). A review of recurrent neural networks: LSTM cells and network architectures. Neural Comput..

[32] Chalapathy R., Chawla S. (2019). Deep learning for anomaly detection: A survey. arXiv.

[33] Tariq S., Lee S., Shin Y., Lee M.S., Jung O., Chung D., Woo S.S. Detecting anomalies in space using multivariate convolutional LSTM with mixtures of probabilistic PCA. Proceedings of the 25th ACM SIGKDD International Conference on Knowledge Discovery & Data Mining.

[34] Chen J., Pi D., Wu Z., Zhao X., Pan Y., Zhang Q. (2021). Imbalanced satellite telemetry data anomaly detection model based on Bayesian LSTM. Acta Astronaut..

[35] Graves A., Mohamed A.r., Hinton G. Speech recognition with deep recurrent neural networks. Proceedings of the 2013 IEEE International Conference on Acoustics, Speech and Signal Processing.

[36] Beirlant J., Goegebeur Y., Segers J., Teugels J.L. (2004). Statistics of Extremes: Theory and Applications.

[37] Siffer A., Fouque P.A., Termier A., Largouet C. Anomaly detection in streams with extreme value theory. Proceedings of the 23rd ACM SIGKDD International Conference on Knowledge Discovery and Data Mining.

[38] Li J., Di S., Shen Y., Chen L. FluxEV: A fast and effective unsupervised framework for time-series anomaly detection. Proceedings of the 14th ACM International Conference on Web Search and Data Mining.

[39] Wang Y., Han Q., Zhao G., Li M., Zhan D., Li Q. (2022). A deep neural network based method for magnetic anomaly detection. IET Sci. Meas. Technol..

[40] Hamoudi M., Quesnel Y., Dyment J., Lesur V. (2011). Aeromagnetic and marine measurements. Geomagnetic Observations and Models.

[41] Tatbul N., Lee T.J., Zdonik S., Alam M., Gottschlich J. Precision and recall for time series. Proceedings of the 32nd Conference on Neural Information Processing Systems (NeurIPS 2018).

[42] Jacob V., Song F., Stiegler A., Rad B., Diao Y., Tatbul N. (2020). Exathlon: A benchmark for explainable anomaly detection over time series. arXiv.

[43] Fawcett T. (2006). An introduction to ROC analysis. Pattern Recognit. Lett..

[44] Hyndman R.J., Koehler A.B. (2006). Another look at measures of forecast accuracy. Int. J. Forecast..

[45] Makridakis S., Andersen A., Carbone R., Fildes R., Hibon M., Lewandowski R., Newton J., Parzen E., Winkler R. (1982). The accuracy of extrapolation (time series) methods: Results of a forecasting competition. J. Forecast..

[46] Noriega G. (2011). Performance measures in aeromagnetic compensation. Lead. Edge.

[47] Noriega G. (2014). Aeromagnetic compensation in gradiometry—Performance, model stability, and robustness. IEEE Geosci. Remote Sens. Lett..

[48] Kumar S., Jain Y.K. (2013). Performance evaluation and analysis of image restoration technique using DWT. Int. J. Comput. Appl..

[49] Jothimani D., Shankar R., Yadav S.S. (2016). Discrete wavelet transform-based prediction of stock index: A study on national stock exchange fifty index. arXiv.

[50] Hardwick C. (1984). Important design considerations for inboard airborne magnetic gradiometers. Geophysics.

[51] Gu B., Li Q., Liu H. Aeromagnetic compensation based on truncated singular value decomposition with an improved parameter-choice algorithm. Proceedings of the 2013 6th International Congress on Image and Signal Processing (CISP).

